# Structured medication reviews in Parkinson’s disease: pharmacists’ views, experiences and needs – a qualitative study

**DOI:** 10.1177/20420986241237071

**Published:** 2024-04-30

**Authors:** Nicol G. M. Oonk, Lucille D. A. Dorresteijn, Eline te Braake, Kris L. L. Movig, Job van der Palen, Henk-Willem Nijmeijer, Mirjam E. van Kesteren, Christina Bode

**Affiliations:** Department of Neurology, Medisch Spectrum Twente, PO Box 50000, Enschede 7500 KA, The Netherlands; Department of Behavioural, Management and Social Sciences, University of Twente, Enschede, The Netherlands; Department of Neurology, Medisch Spectrum Twente, Enschede, The Netherlands; Department of Psychology, Health and Technology, University of Twente, Enschede, The Netherlands; Department of Clinical Pharmacy, Medisch Spectrum Twente, Enschede, The Netherlands; Department of Epidemiology, Medisch Spectrum Twente, Enschede, The Netherlands; Section Cognition, Data and Education, University of Twente, Enschede, The Netherlands; Department of Neurology, Ziekenhuis Groep Twente, Almelo, The Netherlands; Department of Neurology, Isala klinieken, Zwolle, The Netherlands; Department of Psychology, Health and Technology, University of Twente, Enschede, The Netherlands

**Keywords:** medication review, Parkinson’s disease, pharmacy, polypharmacy, primary health care, qualitative research

## Abstract

**Background:**

Executing structured medication reviews (SMRs) in primary care to optimize drug treatment is considered standard care of community pharmacists in the Netherlands. Patients with Parkinson’s disease (PD) often face complex drug regimens for their symptomatic treatment and might, therefore, benefit from an SMR. However, previously, no effect of an SMR on quality of life in PD was found. In trying to improve the case management of PD, it is interesting to understand if and to what extent SMRs in PD patients are of added value in the pharmacist’s opinion and what are assumed facilitating and hindering factors.

**Objectives:**

To analyse the process of executing SMRs in PD patients from a community pharmacist’s point of view.

**Design:**

A cross-sectional, qualitative study was performed, consisting of face-to-face semi-structured in-depth interviews.

**Methods:**

The interviews were conducted with community pharmacists who executed at least one SMR in PD, till data saturation was reached. Interviews were transcribed verbatim, coded and analysed thematically using an iterative approach.

**Results:**

Thirteen pharmacists were interviewed. SMRs in PD were considered of added value, especially regarding patient contact and bonding, individualized care and its possible effect in the future, although PD treatment is found already well monitored in secondary care. Major constraints were time, logistics and collaboration with medical specialists.

**Conclusion:**

Although community pharmacist-led SMRs are time-consuming and sometimes logistically challenging, they are of added value in primary care in general, and also in PD, of which treatment occurs mainly in secondary care. It emphasizes the pharmacist’s role in PD treatment and might tackle future drug-related issues. Improvements concern multidisciplinary collaboration for optimized SMR execution and results.

## Plain language summary

Structured medication reviews in Parkinson’s disease: pharmacists’ views, experiences and needs

Why is this research done?

In Parkinson’s disease (PD), drug therapy is still the most important treatment strategy. Due to disease progression, patients often face complicated medication regimens, polypharmacy, and potential drug-related problems. The execution of structured medication reviews (SMR) in primary care is considered standard care of community pharmacists in the Netherlands, aiming to optimise drug treatment. Although it might also affect clinical outcomes, we found no effect of an SMR in PD on quality of life in our previous study. In trying to improve case management of PD, we need to understand if and to what extent SMRs in PD patients are of added value in the pharmacist’s opinion, and what are assumed facilitating and hindering factors.

What did the researchers do?

We conducted semi-structured interviews with 13 community pharmacists who recently executed ⩾1 SMR in PD.

What did the researchers find?

We found that SMRs in PD are considered of added value with regard to patient bonding and individualised care. By being known by the pharmacist, and vice versa, by knowing the patient’s situation, future drug problems might be tackled earlier. However, executing SMRs comes with barriers, of which lack of time, logistic constraints and difficulties in cooperation with the medical specialist are the most important.

What do these findings mean?

Taking into account both the pharmacist’s effort and additional costs when performing an SMR in the current setting, the valuable time of a pharmacist could potentially better be spent on more (cost-)effective interventions, or a structural solution should be sought for the experienced hindering factors. Since we do not doubt the importance of periodic medication optimization in complex diseases or high-risk patients, we have to focus on either improving the current setting of SMRs in PD, or searching for other strategies in which this can be achieved.

## Introduction

Parkinson’s disease (PD) is among the most common progressive, neurodegenerative diseases with an increasing worldwide incidence.^1^ PD symptoms worsen, among others, due to a slowly progressive shortage of dopamine in the brain by neurodegeneration. Well-known motor symptoms are tremor, rigidity, bradykinesia and postural instability. However, there is a wide spectrum of other impeding motor and non-motor symptoms, which highly influence quality of life.^2–4^ Treatment consists of pharmacotherapy, in which dopamine replacement medicines play a major role. During disease progression, there is a need for higher dosing, more different drugs and more dosing times daily, leading to complex medication schedules. Furthermore, patients often also use medicines for comorbidities, so keeping track of the drug schedule might become challenging.

The complexity of PD and its treatment suggests that a medication review might be valuable: a structured, critical examination of a patient’s medicines aiming to reach an agreement with the patient about therapy, optimizing the impact of medication, minimizing the number of drug-related problems (DRPs) and reducing waste.^5^ The format of a medication review varies from prescription reviews to extensive patient-centred, structured clinical medication reviews.^5^ Medication reviews have been the topic of many studies in the past years and have been shown to reduce potential DRPs and optimize therapy adherence.^6–8^

In the Netherlands, the execution of the abovementioned structured medication reviews (SMRs) by community pharmacists is part of standard care and is performed in close collaboration with the general practitioner (GP). The development of a Systematic Tool to Reduce Inappropriate Prescribing (STRIP) gave guidance for structure and implementation.^9^ However, defining selection criteria for patients for whom an SMR will be most effective has appeared difficult. Since 2014, patient selection criteria for SMRs have changed repeatedly, ranging from selection strictly based on age, number of chronically used drugs and risk factors, to a selection based on degree of vulnerability.^10^ Furthermore, different numbers of SMRs were required to be conducted per pharmacy yearly, which appeared a substantial burden for implementation in primary care.

Our recent study, focusing on the effect of a community pharmacist-led SMR on quality of life in patients with PD, demonstrated that the execution of an SMR appeared challenging in some patients.^11,12^ In this multicentre randomized controlled trial, half of 202 PD patients with polypharmacy received an SMR, while the other half received usual care. Although the process of patient selection was already accomplished and pharmacists had followed training regarding PD, some of the PD patients in the SMR group did not receive an SMR eventually, and reasons for cancelling were not always known. Several factors might influence the practical application and quality of SMRs. The amount of PD patients in a single community pharmacy is relatively small and, therefore, also the exposure of pharmacists to PD. Furthermore, since PD is mainly treated in secondary care by the neurologist, the GP might be less involved. It would, therefore, be of great interest to examine this SMR process from a pharmacist’s perspective.

Two studies explored both the GP’s and pharmacist’s experiences regarding the execution of medication reviews and found resource and time constraints to be important barriers.^13,14^ Also, software difficulties and incomplete medical files were mentioned.^15^ However, these studies were executed in a primary care patient population and thereby lacked a specific focus on PD.

In PD, it has been demonstrated that a multidisciplinary treatment within a network of specialized healthcare providers improves the quality of care.^16,17^ The recognition of the role and contribution of (community) pharmacists in this context is gradually increasing.^18,19^ Yi *et al.*^19^ investigated the involvement of pharmacists and the impact of pharmacy interventions in PD in a systematic review. Their study demonstrated positive effects of pharmacy services regarding the management of DRPs and drug adherence. In order to fully comprehend and facilitate the role of the community pharmacist in PD care in the Netherlands – considering the challenges in the execution of SMRs in PD in our previous study – it seems crucial to obtain more insight into the pharmacists’ perspective on this subject. Their experiences and thoughts shape their decisions. A better understanding of what pharmacists could add to PD care and the experienced bottlenecks in achieving this are the first steps towards further enhancing pharmaceutical care in PD. This pertains to both the SMR process and the PD case management and might thereby shape future research directions and clinical practice.

With this study, we aim to obtain a more profound comprehension of the potential added value, barriers and facilitating factors regarding the execution of community pharmacist-led SMRs in PD, using in-depth semi-structured interviews.

## Methods

### Study design and participants

A cross-sectional, qualitative study was conducted, consisting of face-to-face semi-structured interviews. Participating pharmacists all contributed to the Medication Review in Parkinson study.^12^ A purposive sampling method was used for participant selection with a range of working experience and different pharmacy chain stores. In order to be eligible, participants had to have executed at least one SMR in the study.

In the Medication Review in PD study, pharmacists from 82 pharmacies cooperated and 99 SMRs were performed. Pharmacists were offered accredited training with regard to PD, its pharmacological treatment and a uniform approach to performing SMRs based on the STRIP procedure.^9^ This procedure contains six steps:

1. *Preparation*: The patient is selected and data are collected regarding medication schedule, medical condition, history and last physical examination and laboratory results;
2. *Anamnesis*: Actual medication use is discussed with the patient, including the patient’s views, concerns and expectations;
3. *Analysis*: Medication use is reviewed regarding DRPs, side effects, overuse or underuse, dosing, contraindications, interactions and practical intake problems;
4. *Discussion with physicians*: A pharmacotherapeutic care plan is discussed with involved physicians (GP and/or medical specialist);
5. *Discussion with the patient*: The care plan is discussed with the patient and shared decisions are made regarding drug (use) modifications;
6. *Evaluation*: The pharmacotherapeutic care plan will be monitored within 4 months.

Pharmacists and GPs spent on average 101 min [standard deviations (SD) 57] and 16 min (SD 15), respectively, on an SMR, based on estimation by the involved pharmacist.

### Procedure and data collection

Three central interview topics for examination of potential added value, barriers, and facilitating factors regarding the execution of SMRs were identified.

1. Views and perspectives about the procedure and guidelines regarding the execution of SMRs;
2. The extent to which pharmacists judge themselves capable of executing the SMRs in PD;
3. The value of the PD training beforehand.

An interview scheme with open-ended questions was formulated (Supplemental Data 1). All interviews were audio-recorded and conducted till no new information was obtained. The interviews were carried out face-to-face by one researcher (NO) between October 2018 and February 2019.

### Data analysis

**Figure 1.**
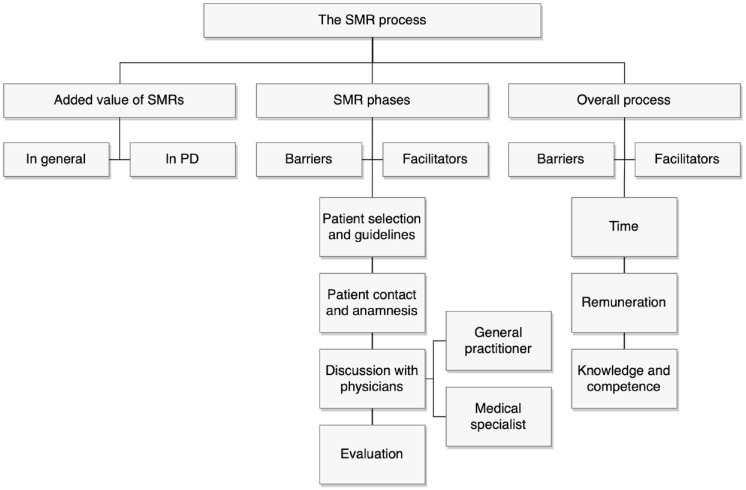
Coding tree. PD, Parkinson’s disease; SMR, structured medication review.

Interviews were transcribed verbatim and coded using ATLAS.ti version 8.4.4 (ATLAS.ti Scientific Software Development GmbH). Subsequently, thematic analysis was carried out based on a constructivist paradigm.^20,21^ After the researchers became acquainted with the data, initial codes were generated. These were reconstructed into themes and subthemes until a saturation point was reached, meaning that no new codes emerged. In this iterative coding process, both inductive and deductive themes were developed.^22^ Deductive coding was based on the different SMR phases of the STRIP protocol and a few a priori considered themes, and additional inductive codes emerged from the verbal material. Finally, a coding tree was developed during the coding of the first six interviews by two researchers and adjusted by discussion with the research team (Figure 1). Subsequently, one of the interviews was independently coded. Inter-rater reliability was assessed by Krippendorff’s α, with α = 0.667 as lowest acceptable limit.^23^ With a coefficient of 0.79 in our study, reliability was secured, and the following interviews were coded separately by the first and second authors.

Three main themes emerged from the interview data: the value of SMRs; factors influencing the execution of SMRs in different SMR phases; and factors influencing the overall process of executing SMRs. The last two mentioned contain both barriers and facilitating factors. Furthermore, influencing factors specifically related to the study setting were explored.

Demographic data were analysed using SPSS version 25. Normality of the data was visually examined. Continuous data are presented as means with SDs when normally distributed or as medians with interquartile ranges alternatively. The reporting of this study conforms to the Standards for Reporting Qualitative Research guideline.^24^

## Results

Thirteen pharmacists were interviewed until data saturation was reached. The mean interview duration was 36 min. Demographic characteristics are shown in Table 1. The mean age is slightly lower compared to the general Dutch community pharmacist population (38.8 *versus* 44.5 years).^25^ More female than male community pharmacists participated. This is in line with the strong age-dependent feminization of the profession.^25^ The mean patient population of a community pharmacy in the Netherlands was on average 8000 in 2019, which is lower than the average in this study.^26^

**Table 1.** Demographic characteristics.

Characteristics	Pharmacists (*n* = 13)
Sex	
Male, *n* (%)	2 (15)
Female, *n* (%)	11 (85)
Age, years	38.8 (9.0)
Practice, years	13.7 (8.5)
Number of yearly executed SMRs	55 (27.4)
Study related^a^	
Executed SMRs	2.0 (0.8)
Mean time spent on SMRs, minutes	92 (42)
Attending accredited PD training, *n* (%)	8 (62)
Pharmacy related	
Mean number of patients per pharmacy	11,985 (4484)

Data are presented as mean ± SD, unless otherwise noted.

a Medication Review in Parkinson study.^12^

SMR, structured medication review.

### The value of SMRs

#### The value of SMRs in general

All participating pharmacists considered an SMR of added value, although the extent of added value and its reasons differed. Mostly mentioned was that it gives a pharmacist better insight into a patient’s functioning, which facilitates a more individualized assessment, and more awareness of potential future problems.

You get a better impression of who the patients are, and how their situation is. Subsequently, you are able to better respond with regard to the treatment they get and the medication they use, and whether or not this is still appropriate. (Pharmacist (Ph) 5)

In line with this, pharmacists reported that the execution of an SMR creates a better relationship with the patient. The contact and attention make future contact with the pharmacy more easily approachable for patients.

There is a lower threshold for patients to pass by or to call when they experience health complaints or side effects. (Ph6)

Also, it was noticed a few times that patients benefit from practical changes and tips regarding drug intake and seem more conscious of their medication afterwards. With regard to the added value in tracing DRPs, the opinions differ. Three pharmacists think an SMR often reveals side effects.

The added value of an SMR is mainly the detection of suboptimal therapy adherence and side effects. And also the fact that patients are afraid of getting side effects. (Ph2)

However, according to six others, the amount of DRPs to tackle is only small.

If you always critically check ‘What medication do I deliver to a patient? Does it interact? Do I give the right amount?’, then you intercept a lot already. (Ph1)

One pharmacist reported that, although valuable, the added value of SMRs in general is less than hoped.

It takes a lot of effort. I need three SMRs, which means three years, to see any change in a patient. [. . .] Patients want SMRs, but do not always know what it comprises. They have a certain expectation, but they also get extra information regarding their drugs by which they need to change their behaviour; that takes time. (Ph11)

#### The value of SMRs in PD

Although thought important, it was questioned by most participants whether SMRs in PD are of more added value than SMRs in other patient groups. Since patients are already strictly monitored by their neurologist, not much was found left to improve in that area. Many participants, therefore, thought that the pharmacist’s contribution to SMRs in PD mainly regards the review of medication, other than anti-Parkinson medication.

When you only look at the anti-Parkinson medication, I think our contribution as pharmacists will not be very large. The other medication, which is beyond the medical specialist’s responsibility, that is where a pharmacist could add something. (Ph12)

### Influencing factors in SMR phases

#### Phase I: Patient selection and guidelines

Although four pharmacists agreed that a certain framework needed to be set for the selection of patients for SMRs, 90% was (simultaneously) critical regarding the specific criteria. Three main barriers were mentioned by most of the interviewees. First, it leads to the same selection of patients yearly, which also comprises patients who are not always motivated or (physically or mentally) capable of an SMR anymore. Second, vulnerability is not completely captured, and an approach addressing prevention is missed. A given solution was to base patient selection on the assessment of GP and pharmacist. Third, the requirement of a certain number of executed SMRs per pharmacy per year is criticized. A more appropriate focus would be on SMR quality instead of quantity.

(1) ‘*I think it is somewhat impeding that only the same patients got reviewed for already 3 or 4 years. [. . .] It feels as an obligation to talk to these patients every year again, while I think: “How much is there still to be achieved?”*’. (Ph7)

(2) ‘*According to the current criteria, eligible patients need to be 65 years or older, whereas I think: “A 40-year old person might also have serious illnesses, or lots of medication. If you guide this patient more carefully at a younger age already, you obtain more profit in the longer term”*’. (Ph11)

(3) If SMRs are not compulsory anymore, ‘*It depends a little on a pharmacist’s intrinsic motivation whether or not SMRs will still be executed*’ (Ph3), but also on the contrary: ‘*There is no need to set a specific number; SMRs are part of your job, so you fix it. Period*’. (Ph11)

A facilitating factor in patient selection is the use of a selection tool program by almost all participants, which could incorporate the applied selection guidelines. Nevertheless, patient selection is still an effort for some.

So far it was well manageable [to achieve the set amount], although the patients where actually important results could be achieved, received an SMR already. I just started [patient selection] for this year. It is difficult to really get something out of it [the SMR]. The search for relevant patients to include is actually most of the effort. (Ph13)

Not related to selection criteria, but another factor pharmacists consider, is the attention that needs to be paid regarding whether patients with certain insurances reached their annual out-of-pocket limit for health care costs already.

I save those patients for later on in the year, since you can better see whether their out-of-pocket limit is already reached. When this is not reached yet, you have to explain to them that they need to pay the costs themselves. [. . .] So I take care not to review these patients in January already. (Ph12)

#### Phase II: Patient contact and anamnesis

Contact with a patient was overall experienced as good, and the common opinion is that patients value a pharmacist’s interest. An important contributor to the quality of the process is a face-to-face conversation, either in the pharmacy or at a patient’s home, compared to dialogues by phone. Patient contact intensifies, and a better assessment could be made with regard to drug adherence.

You will get a lot more out of body language, compared to a conversation only by phone. And it is also good for the patient contact. If you only talk with a patient by phone, you still do not know who this patient is when he or she subsequently visits the pharmacy’. (Ph5)

Still, also in face-to-face communication, estimating a patient’s adherence is thought to remain difficult.

Through specific questions you might get some information, but it will always be superficial. People will not say: ‘I do not take it [the medication] at all’. (Ph13)

Besides, participants agree that these conversations are far more time-consuming than a phone call. Therefore, some prefer to perform the SMR by phone.

I once started inviting people here, but that is dramatic with regard to the time investment. It is really too long for the time I can spend. It is an option that I come to visit, but basically, I tell that the SMR is by phone. That works out very well often. And due to the letter [that patients receive beforehand, with extra information], I notice that people think: ‘Oh, that is what the pharmacist calls for’. (Ph9)

In addition to these facilitating factors, hindering factors were also reported. Although most patients are willing to receive an SMR, participants experience that the cooperation of patients might sometimes be a bit disappointing. This is due to the fact that patients do not have interest in their medication, or are not willing to change.

Sometimes, people are not open to changes. [. . .] Many people have no idea which drug is used for which indication. Some patients are really well informed, [. . .] but many patients are not interested in their medication. They say: ‘I just take this drug, since the doctor wants me to’. (Ph4)

The sometimes-presented reluctance towards a pharmacist’s proposed drug changes might partly be attributed to more reliance by the patient on the physician, compared to the pharmacist.

People often still hold their GP in high esteem: ‘The GP prescribed it, so I need it’. [. . .] Patients do not often see their pharmacist, and that pharmacist is then going to say: ‘This [drug] is stopped’. They prefer to hear that from a GP. It might sometimes be a problem that patients do not want to change for that reason. (Ph13)

According to one participant, this is worse when patients are treated by a medical specialist.

Lastly, 5 of the 13 pharmacists mentioned reaching the patient was difficult.

It starts with planning; this already takes a lot of time. We call the patients ourselves, but often they are not at home, they do not call back, or they are not available. (Ph8)

#### Phase III: Discussion with physicians

##### Discussion with GPs

Contact and discussion with GPs is by nearly all pharmacists experienced as good. Most GPs are willing to be consulted; they are constructive and take proposals seriously. The consultation with a GP is of added value, since GPs know a patient’s history, so more individualized choices can be made.

The GP might know something practical, or has some extra patient background information, and says: ‘That [proposed drug modification] is a good consideration and I would normally agree, but that would not apply for this patient’, because of a specific reason. (Ph12)

Two aspects have been raised as barriers. First, six pharmacists mentioned consultation difficulties when it concerns medication previously started by a medical specialist and not by the GP.

I thought that certain suggestions were quite easily rejected with: ‘That is medication from the medical specialist, I did not start it’. I might find that difficult sometimes. I mean, when you repeat these prescriptions, you also have a certain responsibility. Or you should at least have well-substantiated why the medical specialist would still be responsible. (Ph10)

Second, making a timely appointment can be challenging, according to four participants.

What might be a bit of a bottleneck, is that you need an appointment with the GP on time, to also keep the patient involved. You do not want three months passing, before you finally can call [the patient] again to say: ‘Well, then and then we discussed this’. (Ph6)

One of the GPs had a polypharmacy assistant responsible for scheduling the appointments with both GP and patients, thereby solving this problem. The involved pharmacist mentioned good cooperation.

##### Discussion with medical specialists

A vast majority experienced it as difficult to consult a medical specialist. Several of them sense a threshold. However, if it pertains to something crucial, all participants will seek contact, either by themselves or *via* the GP. The common opinion is that communication with medical specialists is more time- and energy-consuming than with the GP. This was firstly addressed by the fact that specialists are more difficult to reach.

You always get the general reception first, then the ‘secretary of . . .’, and they will write down your question. You eventually hope to have an answer within a week. (Ph1)

Also, participants experience that medical specialists are more reluctant to proposed modifications, and it is frequently unclear whether or not proposed modifications are actually considered.

With a medical specialist, it is more often like ‘Well, we will think about it’, or ‘We will discuss it during the next consultation’. It often remains a bit more vague [. . .]. It is also unclear to me whether anything is done with it [the proposed modification] then. (Ph8)

A few participants mentioned some good experiences with contact by e-mail.

With the medical specialists I always try to e-mail, and it differs a lot per specialist whether you get any reaction. Often I do, I have to say. So I experience that therefore as positive. They seem also positive towards it, since you often actually get a thank you [. . .]. It is only difficult when they do not respond. What to do then, since they are very difficult to reach? However, in general, I think it is positive. (Ph4)

#### Phase IV: Evaluation

In this analysis, interviewee answers regarding the ‘discussion with physicians’ and ‘evaluation’ were sometimes overlapping. However, since these phases are distinguished in the STRIP protocol, this classification was maintained.

The majority of the participants did not mention any barriers according to the evaluation phase. Positive experiences included that concrete arrangements are made regarding the implementation of proposed drug modifications, and tasks are set between GP and pharmacist, which often works well.

At some point, I want to complete it [the SMR], and then I also want to know what happened with it [the proposed modifications]. From that perspective, the requirement to register [the SMR] is actually good, since you keep track of whether anything is actually done with it [the proposed modifications]. (Ph7)

Pharmacists explained to set reminders for checking with the patient how the modifications work out. If the GP was arranged to be in the lead of the follow-up, some would also check with the GP or the GP’s assistant whether or not modifications were implemented, and why.

On the contrary, a few pharmacists experience the follow-up phase as a main bottleneck.

The follow-up, the evaluation, that is a barrier. You register it all well, and often you see that the GP also implemented modifications, but I think this could be better. (Ph2)

### Influencing factors in the overall SMR process

The following influencing factors add to the former results. It pertains to general factors, not related to any of the SMR phases, which influence the overall SMR process.

#### Time

All participants agreed on the fact that an SMR is particularly time-consuming, and time investment is often mentioned as one of the main barriers. This is partly explained by the fact that different steps need to be taken, with different people involved, in which every next step depends on the previous one.

If I do it well, I invite the patient. [. . .]. That is presumed to take 30–45 minutes, but often takes one hour. Then you have to consult the GP, and you subsequently need to inform the patient. This might take 2 hours per patient. Two [hours] times 60 [patients] means 120 hours. It might not sound as that much on a yearly basis, but you nevertheless get tangled. (Ph11)

To structurally organize and plan SMRs is therefore found difficult by at least half of the participants.

A bottleneck is definitely every day’s humdrum. It is very easy to be just busy with your prescriptions, and the questions of the pharmacy technicians and patients. With this, it is difficult to structurally plan time for SMRs. (Ph3)

Besides, the administration coming with an SMR is experienced as time-consuming.

There are some impediments, for example, the administrative process. It [the results of the SMR] has to be registered in the patient file, but also in the health care portal, and subsequently it has to be invoiced to the health care insurance company. (Ph9)

#### Remuneration

Although not directly seen as a barrier, remuneration seems to be an issue. Almost all participants strongly agreed on the fact that the general remuneration for executing SMRs is not sufficient compared to the time it takes, and some wondered whether the distribution of remuneration between the collaborating physicians is fair.

That is obviously not in proportion. Certainly these €30; that is a pittance. You cannot even have a 30-minute conversation for that. (Ph5)

We have to deal with it, but actually, it is not really fair regarding the work it takes. GPs get the same compensation, I think, or even more, although I have the impression that the work is merely done by the pharmacist. (Ph13)

A few participants were not unambiguously negative regarding compensation, by calling it ‘*a good start*’ (Ph9), by classifying it as ‘*part of the job*’ (Ph7), or by the assumption that the rest of the costs will be indirectly compensated *via* other routes.

It regards patients in whom you modify a lot of drugs anyway, and with whom you are busy anyway, so it might in the end be paid back in another way. (Ph1)

One participant had no opinion about the height of remuneration.

#### Knowledge and competence

Regarding knowledge and competence, all participants wanted to express their opinions. Overall, pharmacists feel competent in executing SMRs, although some SMRs – regarding complex disorders or drugs – might be more difficult, for example, psychotropic drugs, extended combinations of inhaled drugs, specific cardiovascular combined drugs, cancer medication and patients with critical renal failure. PD was generally also seen as a complex disease, among others because of the many and different medicines, and the fact that its prevalence in a general community pharmacy is not that high to become very experienced. Participants pointed out that preparatory exploration and delving into PD is required, but all felt capable to a greater or lesser extent.

It [an SMR] could always be better regarding knowledge of the disease and such. However, if I would not have found myself competent, I would not have executed the SMR. I did have a consultation with my colleague before I went to the physician and before I got back to the patient, as in: ‘I found this and this, am I overlooking anything?’. Just as a feedback moment, which I also might do with other SMRs. (Ph10)

In line with this, a few pharmacists explained the responsibility they feel in delivering the right medication.

Even if it is complex, we are still responsible. You cannot think ‘I do not really get it, so I will just distribute it’. [. . .] I want to understand why certain medication is prescribed. [. . .] When you review someone’s medication and you think of a particular drug: ‘What was with this drug again exactly, since I do not come across this one often?’, then you have to find that out first. [. . .] In my opinion, if you distribute drugs, you should know how these work, otherwise you should not do that. (Ph12)

However, nearly all pharmacists are reticent in changing or proposing changes to PD medication prescribed by the neurologist.

I have the feeling that my knowledge about PD is less compared to such a medical specialist. There is a difference in that level. I think it is therefore that we say: ‘Well, let’s keep the PD medication like this, and have a look at the other medication’. Since it is complex. (Ph11)

One participant questioned whether neurologists might even appreciate a pharmacist’s proposed medication modifications regarding PD drugs.

The PD training offered to the pharmacists beforehand was seen as practical and clarifying. Mainly appreciated was the insight provided into the different combinations of PD drugs and their ways of release.

The explanation of why you use a direct release [drug], why you start continued release at a certain moment or another moment; those specific explanations I found very useful. (Ph10)

## Discussion

This is the first study analysing the process of executing SMRs in PD patients from a community pharmacist’s point of view. Insight into the facilitating and hindering factors provides opportunities to improve the SMR process. The key results are discussed below.

SMRs are found valuable, and an important factor concerns patient contact, for both pharmacist and patient. It is thought to lead to better recognition of the pharmacist as healthcare provider. Furthermore, by knowing a patient’s situation and expectations, more individualized and shared decisions could be made regarding a treatment plan, so future problems might be tackled earlier. A qualitative study regarding barriers and facilitators of implementation of SMRs for general primary care patients supports our findings: both the relationship with the patient and the interprofessional relationship with GPs improved.^14^

Specifically regarding PD, the added value was mentioned to be in practical drug intake problems that might occur with disease progression and the spectrum of comorbidities in PD for which also other drug treatment might be needed. The pharmacists were somewhat reticent to modify PD medication, among others due to frequent monitoring of these drugs by the neurologist. However, in our analysis regarding proposed drug interventions, classified by 12 drug classes, 19% concerned anti-dopaminergic drugs, which was thereby the biggest category.^12^ This different finding might be explained by the fact that the pharmacist was possibly more than usually focused on PD drugs since PD patients were the target of our study. It is, however, not *per se* a problem when the pharmacist prefers not to change the medical specialist’s prescriptions: the pharmacist takes into consideration the entire spectrum of used drugs, including practical aspects regarding drug intake. Nevertheless, it demonstrates room for improvement regarding multidisciplinary cooperation, in such a way that the neurologist and pharmacist collaboratively assess opportunities for drug optimization. Although reviewing drugs of patients with a complex disease, pharmacists in general felt capable of this task.

One of the main hindering factors in executing SMRs was lack of time. SMRs are time-consuming due to all the steps that need to be prepared, planned and checked, and the fact that at least three or more people are involved. Time constraints were mentioned in other research, where SMRs were even executed without patient involvement, due to high workload and competing priorities.^13,15,27–29^ It also became one of the barriers to deprescribing medicines.^13^ Furthermore, in a New Zealand study, 37% of the invited pharmacists declined to participate, with time issues as the main reason.^30^ In the same study, more than 60% of the pharmacists who initially agreed to participate, did not start the study eventually or started without final completion with again time as the most important barrier.^30^ Although withdrawal rates in our previous study were lower, there were also PD patients who did not receive an SMR despite commitment of the involved pharmacist.^12^

A second specific barrier was the experienced difficulty in consulting medical specialists and the lack of feedback. This is noteworthy, since GPs are also hesitant to change prescriptions that originate from a medical specialist in secondary care.^15^ The added value of medical specialists being involved in the SMR process but also the concerns around communication were also raised by Belgian community pharmacists and by pharmacists carrying out medication reconciliation for recently discharged patients.^14,27^ The importance of interdisciplinary cooperation was further underlined in a medication management study for patients with advanced dementia.^31^ Another part of this barrier is that community pharmacists often lack access to electronic medical records in secondary care. These are important findings, since a better involved medical specialist and adequate information transfer could contribute to the quality of the SMR and to its clinical effect.^32^

The upgrade of the patient selection criteria in 2019 has the potential to make the execution of SMRs more time efficient, among others by relinquishing the previous strict selection criteria.^10^ Although guidance for patient selection is given, the selection is recommended to be primarily based on the assessment of pharmacist and GP. Furthermore, attention is given to patients in secondary care, with a strong recommendation to strive for better involvement of all treating physicians. Our results undoubtedly confirm these conclusions.

Since it is thought important to keep progressively complex drug treatment as optimized as possible, there has become more interest in the role of the community pharmacist within PD care in the last decade.^19^ In our quantitative Medication Review in PD study, executed in accordance with the Dutch Medication Review guidelines at that time, we found no improvement in quality of life in PD patients after an SMR and higher expenditure of health care costs compared to usual care.^9,12,33^ The explanation for these outcomes seems multifactorial. The merely mild disease severity, the primary care setting, the difficult multidisciplinary communication lines, patient characteristics or any combination might play a role.

A systematic review regarding pharmacy interventions in PD found that PD pharmacy services included providing education, tackling DRPs and adherence issues and carrying out medication reviews.^19^ The different study settings and interventions made drawing definite conclusions regarding their impact difficult, although based on their meta-analysis drug adherence seemed to improve. The effect on quality of life was disputable. A Dutch pilot study did find a positive effect on quality of life, after a multi-step intervention of which one comprised an SMR, performed in an outpatient PD clinic by a pharmacist with extended PD expertise.^32^ This setting is however fundamentally different compared to ours and thereby to what was – till recently – applied as standard care.

Although the opinion of the pharmacist was not previously studied, a few studies reflect on pharmaceutical interventions in PD from a patient’s perspective. One study explored the regular involvement of a clinical pharmacist in an outpatient PD clinic with anonymous surveys.^34^ Positive evaluations came from both patients and other healthcare providers. Contributions were made particularly in the field of solving DRPs and providing drug education, thereby leaving more space for healthcare providers to allocate their time to other responsibilities.^34^ Also, patients consistently indicated that the pharmacist’s involvement was either ‘always’ or ‘often’ necessary to impact their treatment, after the implementation of a pharmacist intervention plan.^35^ At last, a recent Malaysian study emphasized the pharmacists’ role in the delivery of pharmaceutical care services in addressing drug challenges faced by PD patients.^36^ However, it was thought essential for the patients to recognize the expanded role of the pharmacist, as was also mentioned by the pharmacists in our study.

### Clinical implications and future perspectives

Our quantitative outcomes, together with the abovementioned qualitative insights, question whether the current SMR approach needs to change. An interesting concept regarding integrated (pharmaceutical) care was implemented by Hazen *et al*.,^37^ who studied whether pharmacotherapy could be optimized when integrating a clinical pharmacist in the general practice. Although this differs from our study regarding focus (general primary care *versus* PD) and setting of care (no multidisciplinary secondary care involved), it showed an efficient way of applying SMRs and pharmaceutical care. By better cooperation, significantly more proposed drug interventions were implemented, and community pharmacists encountered benefits of their colleagues being located at the general practice.^38,39^

With this in mind, a way of addressing (parts of) the constraints regarding time, logistics and multidisciplinary cooperation found in our study might be to centralize PD primary (pharmacist) care by community pharmacists with a special focus on PD treatment, either at their pharmacy or at a PD clinic, like the mentioned Dutch pilot study of Stuijt *et al*.^32^ There are three of those PD focused clinics in the Netherlands nowadays, where all PD patients from the corresponding regions are treated. These centres could enable short communication lines between involved neurologists and community pharmacists, since the multidisciplinary team is better defined. Some of these centres also have a PD-focused clinical pharmacist, who could further bridge pharmacy care. This might relieve other community pharmacists of extra (SMR) workload. Additionally, the pharmacist’s role within PD care might be emphasized for the patient. With this, patients could better acknowledge the pharmacist as an involved partner in their treatment, which might influence the previously described – and also here experienced – reluctance towards drug modifications.^27,28,40^ Also, PD patient selection for SMRs might be optimized, since all collaborating healthcare professionals could keep track of vulnerability.

Apart from a few pilot studies, no data are yet available regarding the effects of centralizing PD pharmaceutical care.^41,42^ Since we can conclude that the current setting of executing SMRs in PD in primary care should change, the potential of executing SMRs in the abovementioned new setting offers interesting options for new research, both quantitatively and qualitatively. Herewith, potential barriers (e.g. logistic problems for less mobile PD patients) and the point of view of both community pharmacists and PD patients should be further studied.

### Strengths and limitations

Among the strengths of this study are the insights into pharmacists’ views regarding the execution of SMRs, not only in PD patients, but also in general. A qualitative point of view adds to the quantitative data and makes a more nuanced interpretation possible. We think the presented results are a good reflection of the general practice regarding the execution of patient-centred SMRs, and the iterative approach with two co-coders analysing the interviews expanded the interpretation of our data by generating unanticipated insights next to expected points of view.

One of the limitations was that two of the interviews had not been fully recorded due to technical problems, and we could not use these missing data. However, since we proceeded with interviewing until no new data emerged, we do not think this eventually influenced our overall results. Another limitation is the fact that the previous applicable patient selection guidelines were updated during the study. However, as previously mentioned, our findings support the changes that have been made in the updated guidelines.

Furthermore, one could question the generalizability of the current results to other Dutch pharmacies, since the interviewed pharmacists were all willing to cooperate in the clinical trial on SMRs in PD, and executing SMRs in a study setting might be different from the normal situation. However, almost no pharmacist refused cooperation, and the demographic data are a good reflection of the average Dutch community pharmacist.^26,43^ Also, when questioning pharmacists, no or only a little difference was experienced: the way of performing SMRs in this study setting, using the STRIP protocol, was in accordance with the existing guidelines and usual practice. The small differences mentioned were the fact that an appointment with the GP was both easier made in a study setting, as well as more difficult. The time span for executing these SMRs was found too short by some participants, and pharmacists had to adjust their own SMR planning and structure, since the intervention group patients in this study came in between.

## Conclusion

To conclude, performing SMRs in PD patients in primary care is considered valuable from a community pharmacist’s point of view, both for direct patient-centred care, and for the future, with the potential to manage future issues at an earlier stage. The most hindering factors for executing SMRs are time and logistic constraints. Also, multidisciplinary collaboration, known as of high importance in PD care, leaves room for optimization.

## Supplemental Material

sj-docx-1-taw-10.1177_20420986241237071 – Supplemental material for Structured medication reviews in Parkinson’s disease: pharmacists’ views, experiences and needs – a qualitative study Supplemental material, sj-docx-1-taw-10.1177_20420986241237071 for Structured medication reviews in Parkinson’s disease: pharmacists’ views, experiences and needs – a qualitative study by Nicol G. M. Oonk, Lucille D. A. Dorresteijn, Eline te Braake, Kris L. L. Movig, Job van der Palen, Henk-Willem Nijmeijer, Mirjam E. van Kesteren and Christina Bode in Therapeutic Advances in Drug Safety

sj-docx-2-taw-10.1177_20420986241237071 – Supplemental material for Structured medication reviews in Parkinson’s disease: pharmacists’ views, experiences and needs – a qualitative study Supplemental material, sj-docx-2-taw-10.1177_20420986241237071 for Structured medication reviews in Parkinson’s disease: pharmacists’ views, experiences and needs – a qualitative study by Nicol G. M. Oonk, Lucille D. A. Dorresteijn, Eline te Braake, Kris L. L. Movig, Job van der Palen, Henk-Willem Nijmeijer, Mirjam E. van Kesteren and Christina Bode in Therapeutic Advances in Drug Safety

## Declarations

**Ethics approval and consent to participate:** This study was performed in line with the principles of the Declaration of Helsinki. Approval was granted by the Medical Ethical Review Board Twente, the Netherlands (reference number NL48661.044.14). Written informed consent was obtained from all individual participants included in the study.

**Consent for publication:** Not applicable.

**Availability of data and materials:** The datasets generated during and/or analysed during the current study are available from the corresponding author upon reasonable request.
