# Supplementary material for: Structured medication reviews in Parkinson’s disease: pharmacists’ views, experiences and needs – a qualitative study
Source: Ther Adv Drug Saf. 2024 Apr 30;15:20420986241237071. doi: 10.1177/20420986241237071 (PMC11062216; doi:10.1177/20420986241237071)
Supplement: sj-docx-2-taw-10.1177_20420986241237071 – Supplemental material for Structured medication reviews in Parkinson’s disease: pharmacists’ views, experiences and needs – a qualitative study [file sj-docx-2-taw-10.1177_20420986241237071.docx]

**Supplementary data 1**

Interview scheme – Structured medication review in Parkinson’s disease

﻿**Start**

Acquaintance and thanking for cooperation, explaining the process, acquiring informed consent, and starting the interview.

**Theme 1: a pharmacist’s view and perspective regarding the execution of structured medication reviews (SMRs).**

1. How useful do you think an SMR is? For example, regarding benefits for the patient (and if so; in which way?), drug related problems, cost reduction, having an up-to-date overview of a patient’s medication use, etcetera.

*The current guideline of the Health and Youth Care Inspectorate regarding SMRs states that minimally 60 (and last year 100) SMRs have to be executed yearly per pharmacy.*

1. What do you think of this guideline?
2. How feasible is this number yearly?
3. Do you achieve this number?
4. How many SMRs do you perform on average yearly?
5. How much time do you spend on average on an SMR?

*In different recent studies, it appears not clear which (high risk) patient groups benefit the most from an SMR.*

1. How are patients selected for an SMR in your pharmacy?
2. Do you do that yourself?
3. Which guidelines are there for patient selection?
4. Could it be that patient selection - whether or not unintentional - is based on your own preferences (e.g. age, certain specific diseases, etcetera)? And if yes; how?

*After patient selection, you contact the patient and obtain an anamnesis.*

1. What is your impression of a patient’s knowledge regarding their medication, therapy adherence, and practical drug intake? Are you often in any way surprised?

*Often discussion with the prescribing physicians occurs afterwards, regarding medication or proposed drug modifications.*

1. How do experience the cooperation with other physicians in general?
2. How is their willingness to cooperate?
3. How do they address your proposed modifications?
4. What are the differences between cooperation with a general practitioner (GP) and a medical specialist?

*Subsequently, the prescribing physician does or does not implement the proposed modification(s).*

1. How is the SMR process continuing for you from that point on?
2. How responsible do you feel for the implementation of proposed modifications?
3. How is checked whether or not certain modifications are definitely implemented when the GP is in charge? Do you check this in any way?
4. Which bottlenecks can you mention in the process of executing SMRs?

*Pharmacists obtain a fixed remuneration from the patient’s health care insurance company for the execution of an SMR.*

1. What do you think of the amount?
2. Is it sufficient?

**Theme 2: the execution of the SMR and the extent to which pharmacists judge themselves competent regarding the execution of SMRs in PD.**

*All community pharmacists should be able to execute SMRs in all different patient groups. However, treatment of specific patient groups might be rather complex or rare, which might make the execution of an SMR more difficult.*

1. Do you recognize this? And if yes; in what diseases or patient groups?
2. Did you execute SMRs in PD before?
3. How do you think the execution of the SMR went in this study setting?
4. How did you prepare?
5. What more would or should you have done regarding the preparation?

*Within the different steps to be taken in the execution of an SMR, you have had contact with the patient (and different health care professionals).*

1. Did you speak with the patient face-to-face?
2. What were your considerations in whether or not having face-to-face contact?
3. How did you experience the contact with the patient?
4. What were the patient’s expectations of the SMR?
5. Were these expectations realistic in your opinion?
6. What would you perhaps do differently next time? Why, and how?
7. To what extent do you think the aim of the SMR was clear for the patient?

*GPs of all patients randomized in the SMR group were informed by letter regarding inclusion and the intended SMR.*

1. How did you experience the contact with other health care professionals?
2. How was the contact with the GP
3. Which other health care providers were involved?
4. How was the contact with those?
5. If you had contact with the neurologist; was this neurologist informed about the study?
6. How was the cooperation with the involved health care professionals?
7. How did they deal with your proposed modifications?
8. What were the reasons for either or not implementing modifications?
9. Which factors were hindering or facilitating the process of executing the SMR? E.g. own knowledge, and knowledge of other health care professionals; reaching the patient; communication with the patient and other health care professionals; structural delays; the responsibility for prescribing medication; the openness to proposed modifications; etcetera.

*We aimed for SMRs to be preferably executed within two weeks. This appeared not feasible in practice.*

1. How did you experience potential time pressure?
2. What do you think is a realistic time span for an SMR to be executed?

*We hypothesized that an SMR in PD might be of high value, due to the often complex medication schedules, the progressiveness of the disease, the often higher age, and comorbidities.*

1. How useful are SMRs in PD to your opinion?
2. What is different in this setting compared to SMRs you normally execute? E.g. the patient population, the disease complexity, own experience, the cooperation with other health care professionals, etcetera.
3. To what extent did you feel capable and competent to execute SMRs in this patient population?

**Theme 3: the contribution of the PD training offered to all pharmacists.**

1. Did you attend the PD training?

If yes:

1. What did you think of it?
2. Was it helpful in executing the SMR?
3. Did it meet your expectations? Why? And if not: what did you miss?

If no:

1. What was the reason for not attending the training?
2. Did you use the hand-outs that were delivered with the training before or during the SMR?
3. What were you looking for, and did you find this?
4. Could we have better prepared you than we did?
5. What did you miss from the research team?
6. Is there anything more that you would like to mention, or do you have any questions left?
